# Prognostic Factors of Platinum-Refractory Advanced Urothelial Carcinoma Treated with Pembrolizumab

**DOI:** 10.3390/cancers15245780

**Published:** 2023-12-09

**Authors:** Yasunori Akashi, Yutaka Yamamoto, Mamoru Hashimoto, Shogo Adomi, Kazutoshi Fujita, Keisuke Kiba, Takafumi Minami, Kazuhiro Yoshimura, Akihide Hirayama, Hirotsugu Uemura

**Affiliations:** 1 Department of Urology, Kindai University Nara Hospital, Ikoma 630-0293, Japan; 144053@med.kindai.ac.jp (Y.A.);; 2 Department of Urology, Kindai University Hospital, Osakasayama 589-8511, Japan

**Keywords:** advanced urothelial cancer, immune checkpoint inhibitor, antibiotic exposure

## Abstract

**Simple Summary:**

Several studies have investigated various types of biomarkers to predict responses to immune checkpoint inhibitor (ICI) therapy for patients with platinum-refractory advanced urothelial carcinoma, but they were inconclusive. Recently, antibiotic exposure has attracted attention as a biomarker because it may affect antitumor immunity through changes in gut microbiota. We evaluated the factors predictive of ICI response, including antibiotic exposure, in 41 metastatic urothelial carcinoma patients. The patients’ median age was 75 years, and the vast majority of the patients were male. The objective response rate was 29.3%, with a median overall survival (OS) of 17.8 months. A high neutrophil-to-lymphocyte ratio (NLR) and poor performance status (PS) were significantly associated with poor OS. Antibiotic exposure did not have a significant impact on OS.

**Abstract:**

Introduction: Immune checkpoint inhibitor (ICI) therapy has significantly improved the prognosis of some patients with advanced urothelial carcinoma (UC), but it does not provide high therapeutic efficacy in all patients. Therefore, identifying predictive biomarkers is crucial in determining which patients are candidates for ICI treatment. This study aimed to identify the predictors of ICI treatment response in patients with platinum-refractory advanced UC treated with pembrolizumab. Methods: Patients with platinum-refractory advanced UC who had received pembrolizumab at two hospitals in Japan were included. Univariate and multivariate analyses were performed to identify biomarkers for progression-free survival (PFS) and overall survival (OS). Results: Forty-one patients were evaluable for this analysis. Their median age was 75 years, and the vast majority of the patients were male (85.4%). The objective response rate was 29.3%, with a median overall survival (OS) of 17.8 months. On multivariate analysis, an Eastern Cooperative Oncology Group performance status (ECOG-PS) ≥ 2 (HR = 6.33, *p* = 0.03) and a baseline neutrophil-to-lymphocyte ratio (NLR) > 3 (HR = 2.79, *p* = 0.04) were significantly associated with poor OS. Antibiotic exposure did not have a significant impact on either PFS or OS. Conclusions: ECOG-PS ≥ 2 and baseline NLR > 3 were independent risk factors for OS in patients with platinum-refractory advanced UC treated with pembrolizumab. Antibiotic exposure was not a predictor of ICI treatment response.

## 1. Introduction

Gemcitabine, cisplatin (GC) and methotrexate, vinblastine, doxorubicin, and cisplatin (MVAC) are widely used as first-line regimens in metastatic urothelial cancer (UC), but many cases are refractory [1]. After the failure of platinum-based chemotherapy, there was no internationally accepted standard of care. However, since the advent of an immune checkpoint inhibitor (ICI) for advanced UC, the treatment strategy for UC patients has changed dramatically. Pembrolizumab is a humanized monoclonal IgG4κ isotype antibody that directly inhibits programmed cell death-1 (PD-1) and its ligands, programmed cell death ligand-1 (PD-L1) and programmed cell death ligand-2 (PD-L2). PD-1 inhibits cytokine production from T cells and cell proliferation by binding to PD-L1, which results in the suppression of immune responses [2]. PD-L1 also suppresses immune responses by converting naïve CD4(+) T cells into regulatory T (Treg) cells [3]. Pembrolizumab inhibits the binding of PD-1 to both PD-L1 and PD-L2 ligands, activating cancer-specific cytotoxic T lymphocytes and the PD-L1 and PD-L2 ligands.

In the randomized phase 3 Keynote-045 trial, pembrolizumab showed better outcomes than chemotherapy as a second-line therapy for UC patients who progressed after platinum-containing regimens [4]. Based on these results, current guidelines recommend pembrolizumab as a second-line systemic therapy after platinum-containing regimens [5]. However, pembrolizumab offers an objective response rate (ORR) of approximately 20%, which is anything but satisfactory. Identification of predictive biomarkers could increase the benefit of ICI treatment and avoid therapeutic intervention if the likelihood of response is predicted to be low. Therefore, it is crucial to identify biomarkers that predict which patients will benefit from pembrolizumab treatment. Promising biomarkers for predicting the response to ICI therapy include the expression of programmed death ligand-1 (PD-L1), the tumor mutational burden (TMB), and circulating tumor DNA in various types of cancer [6,7,8]. However, an ideal biomarker would be reproducible, cost-effective, and simple. From this perspective, the usefulness of the Eastern Cooperative Oncology Group performance status (ECOG-PS), location of the metastasis, C-reactive protein (CRP), and the neutrophil-to-lymphocyte ratio (NLR) has been studied in several malignancies, including UC [9,10,11,12,13,14,15,16]. More recently, one factor that is attracting interest is the association between ICI treatment outcomes and antibiotic exposure. This is due to the fact that antibiotic exposure alters the gut microbiome, which in turn affects the effectiveness and immune-related toxicities of ICIs [17,18,19,20,21,22]. However, few reports have evaluated the association between antibiotic exposure and ICI treatment outcomes of patients with UC [23]. The purpose of this study was to investigate potential predictive biomarkers, including antibiotic exposure, in patients with UC treated with pembrolizumab, which could provide useful information for patients who require ICI treatment.

## 2. Materials and Methods

### 2.1. Patients and Clinical Data

The study cohort consisted of advanced or metastatic UC patients who received pembrolizumab at Kindai University Nara Hospital and Kindai University Hospital in Japan between January 2018 and December 2021. All patients received a pathological diagnosis of UC and were treated with platinum-containing neoadjuvant or adjuvant chemotherapy. All samples enrolled in this study were acquired via surgical resection or biopsy. Clinicopathological data were obtained from the patients’ medical records. Pembrolizumab 200 mg was administered intravenously every three weeks, and it was continued until unacceptable toxicity or either radiographic or clinical disease progression. This study was approved by the Institutional Review Board of Kindai University Nara Hospital and Kindai University Hospital (approval number 705). Informed consent was waived because of the retrospective design of the study. However, an opt-out opportunity for this study was provided through the website of our institution (https://www.med.kindai.ac.jp/uro/, accessed on 5 December 2023).

### 2.2. Statistical Analysis

Progression-free survival (PFS) and overall survival (OS) were calculated with the Kaplan–Meier method. The ORR to pembrolizumab was assessed according to the response evaluation criteria in solid tumors (RECIST) version 1.1 [24]. Adverse events (AEs) following pembrolizumab were evaluated at each visit during and after treatment. The severity of the AEs was graded according to the National Cancer Institute’s common terminology criteria for adverse events (CTCAE) v5.0 [25]. Univariate and multivariate analysis were performed using a Cox proportional hazards model to identify the biomarkers for PFS and OS. A *p*-value < 0.05 was considered significant. The Statistical Package for the Social Sciences version 23.0 (SPSSs, Chicago, IL, USA) was used for all statistical analysis.

## 3. Results

### 3.1. Patients’ Characteristics

A total of 41 patients met the eligibility criteria and were evaluable for this analysis. The median duration of pembrolizumab treatment was 4.0 (0–25.0 PS) months, and the median follow-up period was 16.5 (range, 1.0–47.8) months. No patients received atezolizumab as a second-line therapy after chemotherapy because it was not covered by the Japanese health insurance system. Table 1 shows the patients’ baseline characteristics. The patients’ median age was 75 (range, 58–81) years, and the vast majority of the patients were male (85.4%). Sixteen of the forty-one patients had upper UC. Twenty-nine patients had undergone total cystectomy or total nephroureterectomy, and eight patients had received radiation therapy. ECOG-PS was 0 or 1 in 90.2% (37/41) of patients. The median number of cycles of pembrolizumab treatment was five (range, 1–32). Lymph nodes were the most common sites of metastases (66%, 27/41), followed by the lungs (49%, 20/41), bones (22%, 9/41), and liver (15%, 6/41). The median neutrophil-to-lymphocyte ratio (NLR) at baseline was 2.96 (range, 1.27–28.4). Antibiotic exposure was defined as antibiotic use for at least 1 week within 1 month before or after starting pembrolizumab. Sixteen patients (39%) were identified as the antibiotic exposure cohort, with a median duration of antibiotic exposure of 7 (range, 7–30) days. With regard to antibiotic classes, they included cephalosporins in seven cases, fluoroquinolones in five cases, and penicillins in four cases. The most common reasons for the use of antibiotics were urinary tract infections (24%), followed by other infections (12.2%) or febrile neutropenia (2.4%).

### 3.2. Efficacy of Pembrolizumab and Adverse Events

**Figure 1:**
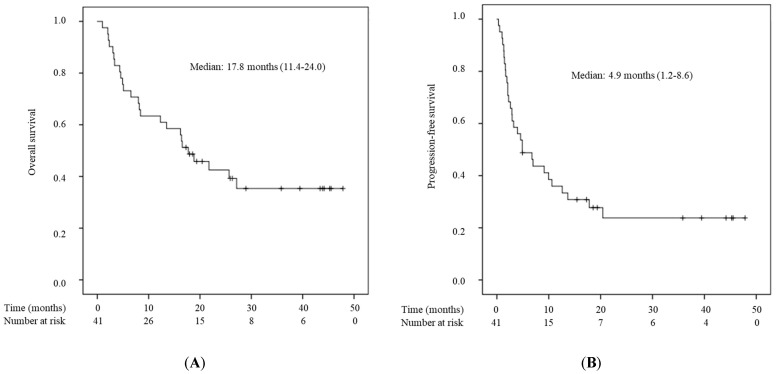
A Kaplan–Meier curve of overall survival (**A**) and progression-free survival (**B**) for 41 patients in the cohort study.

The OS and PFS are shown in Figure 1A,B. Thirty-one patients (75.6%) discontinued treatment with pembrolizumab because of disease progression (70.7%) and immune-related AEs (4.9%). Twenty-five patients (60.9%) had died at the time of analysis. The median PFS and OS were 4.9 [95% confidence interval (CI), 1.2–8.6] months and 17.8 [95% CI, 11.5–24.0] months, respectively. The causes of death of the 25 patients who died were UC in 24 (96%) and suicide in 1 patient (4%). The best overall response assessed according to RECIST is shown in Table 2. Totals of 7 (17.1%), 5 (12.2%), 2 (4.9%), and 27 (65.9%) patients were diagnosed with complete response (CR), partial response (PR), stable disease (SD), and progressive disease (PD), respectively. The ORR was 29.3%. Immune-related AEs were observed in 35 cases (85.4%), of which 5 cases (12.2%) experienced CTCAE grade ≥ 3 AEs (Table 3). The most common treatment-related adverse events of any grade were rash (22% of the patients), hypothyroidism (10%), and interstitial pneumonia (10.9%). One patient was diagnosed with severe interstitial pneumonia. He received treatment with hyperbaric oxygen therapy and high-dose steroid administration but died of respiratory failure (grade 5). One patient experienced grade 4 liver dysfunction and received high-dose steroid administration. Two weeks after steroid administration, the levels of hepatic enzyme were normalized.

### 3.3. Risk Factors for Shorter Survival

As shown in Table 4, univariate analysis showed that ECOG-PS ≥ 2 (*p* = 0.01), baseline NLR > 3 (*p* = 0.01), hemoglobin ≤ 11 g/dL (*p* = 0.01), and lower urinary tract tumor (*p* = 0.03) were significantly associated with inferior PFS. However, factors that predict shorter PFS could not be identified based on multivariate analysis. Also, ECOG-PS ≥ 2 (*p* = 0.01), baseline NLR > 3 (*p* = 0.01), hemoglobin ≤ 11 g/dL (*p* = 0.01), and CRP ≥ 1 mg/dL (*p* = 0.04) were significantly associated with inferior OS. Multivariate analysis, including all significant factors identified on univariate analysis, showed that ECOG-PS ≥ 2 (*p* = 0.03) and NLR > 3 (*p* = 0.04) were significantly associated with inferior OS (Table 5). Antibiotic exposure did not have a significant impact on either PFS or OS.

## 4. Discussion

In this study, pembrolizumab resulted in a median PFS of 4.9 (95% CI, 1.2–8.6) months and a median OS of 17.8 (95% CI, 11.5–24.0) months. These results are slightly better than those of the Keynote-045 trial [4], which reported a median PFS of 2.1 months and a median OS of 10.1 months. In addition, pembrolizumab provided an ORR of 29.3%, higher than in the Keynote-045 trial (21.1%). These results are comparable to those of other retrospective studies evaluating the efficacy and safety of pembrolizumab in advanced UC [26,27,28].

Several studies proposed biomarkers with prognostic value in patients who received pembrolizumab. In this study, ECOG-PS ≥ 2 and NLR > 3 were identified as independent risk factors for a poor prognosis. In a recent meta-analysis, a poor ECOG-PS, the presence of visceral metastasis, and high pretreatment levels of NLR and CRP were associated with shorter OS [13], similar to the present results. ECOG-PS has been widely used as a tool to validate indications for systemic therapy [14], and poor ECOG-PS has been reported to be associated with shorter OS in patients with advanced UC treated with chemotherapy or ICIs [15,16,29]. Conversely, the phase 2 Keynote-052 trial, which evaluated safety and antitumor activity in patients with locally advanced or metastatic cisplatin-ineligible UC, reported that poor ECOG-PS did not have an impact on the efficacy of pembrolizumab [30]. Parikh et al. reported trends in initiating end-of-life systemic therapy in 1637 patients with metastatic UC [31]. They found a significant increase in the use of systemic therapy in patients with poor PS after the approval of ICIs. The toxicity profile of ICIs compares favorably with chemotherapy, which may have increased the opportunity to administer such drugs to patients with poor PS who would otherwise have been on best supportive care (BSC). Our study also resulted in a higher percentage of patients with ECOG-PS ≥ 2 (10%) compared with the Keynote-045 trial [4], which included only 0.7% of patients with ECOG-PS ≥ 2. The reason for this may be that immediately after ICI approval, some patients with poor ECOG-PS who should have been considered for BSC preferred ICI treatment, which has a relatively favorable toxicity profile compared with chemotherapy. However, given the possibility that poor ECOG-PS may lead to shorter OS, whether ICIs should be used in all patients will require further investigation.

The present results also showed that the inflammation-based prognostic marker NLR was significantly associated with OS. There has been controversy over the impact of NLR on the responses to ICI. Previous studies have shown that NLR prior to ICI initiation is associated with survival in patients with metastatic UC [10,11,12,13], and that a high NLR is a potential risk factor for poor clinical outcomes in various malignancies [32]. The mechanism by which NLR is related to the response to ICI treatment and survival is uncertain. Kargl et al. reported that a high pretreatment neutrophil count was associated with a decreased number of CD8-positive T cells, which resulted in reduced antitumor activity [33]. Furthermore, Friedlander et al. reported that a higher NLR with shorter survival is due to an increase in the N2 phenotype of the neutrophils, which play a pro-tumorigenic role [34]. Given the potential of NLR as a predictive biomarker that can be easily used in routine clinical practice, further research is warranted to validate its utility and precise mechanism.

Several studies have examined the impact of antibiotic exposure on the response to ICI therapy, but they were inconclusive, with some reporting a positive relationship and others a negative one [17,18,19,20,21,22,23,35,36,37]. In the present study, antibiotic exposure was not related to PFS or OS in patients on ICI treatment. It has been hypothesized that antibiotic exposure may affect responses to ICI treatment through changes in the gut microbiome [37]. Several potential mechanisms explain the relationship between changes in the gut microbiome and ICI response. Mechanisms that include intestinal dendritic cells, which affect T-cell priming and activation, can be affected by specific bacterial strains, and microbiome metabolites modulate host cytokine production and T-cell responses [38,39]. Gopalakrishnan et al. reported interesting findings using a mouse model of melanoma with different compositions of gut microbiota. They found that tumors grew more rapidly in the group with failing microbiota than in those with favorable microbiota, but the tumor growth could be altered by transferring fecal material from the favorable microbiota groups [19]. Despite these findings, antibiotic exposure affected neither PFS nor OS in the present analysis. The relatively small number of patients analyzed in the present study, the definition of the timing, and the duration of antibiotic exposure may be the reasons why no correlation between antibiotic exposure and prognosis was found. In this study, we defined antibiotic exposure as antibiotic use for at least 1 week within 1 month before or after starting ICI treatment with reference to previous reports [40,41,42,43]. Reports on the association between the duration of antibiotic exposure and changes in gut microbiome are mixed. Dethlefsen et al. reported the changes in the intestinal microbiota of healthy subjects before and after ciprofloxacin administration. They found that ciprofloxacin had a long-term effect on the post-treatment gut microbiota, but the majority of the gut microbiota returned to pre-treatment levels after 4 weeks [44]. Although antibiotic exposure was not an independent risk factor for poor prognosis in our study, it might be a prognostic factor if the duration of antibiotic exposure were defined differently. Furthermore, another factor responsible for the present result may be the type of antibiotic. Eng et al. reported the impact of antibiotic exposure before ICI treatment on OS in 2737 patients with various types of cancer [45]. They found that antibiotic exposure before ICI treatment was associated with worse OS, but this was observed only with fluoroquinolone exposure and not with penicillin or cephalosporin exposure. Fluoroquinolones can alter many gut microbiota species, including Alistipes, Bifidobacteria, Faecalibacterium, and Ruminococcus, which have been found to affect ICI outcomes [46,47,48,49,50]. In the present study, about 70% of cases used penicillins or cephalosporins, which may be the reason why antibiotic exposure was not identified as a predictor for ICI response. Although several studies have examined the association between ICI treatment outcomes and antibiotic exposure, few have examined this issue specifically in UC patients, and to the best of our knowledge, we are the first to report a lack of correlation between them. To clarify antibiotic exposure as a promising biomarker in real-world clinical practice, it will be necessary to determine whether the ICI response varies depending on the type of antibiotic and the duration of antibiotic exposure in future studies.

In addition to clinicopathological data, multiple biomarker studies have evaluated tumor- and tumor microenvironment-related factors associated with the response to ICI therapy [51]. PD-L1 immunohistochemistry remains a controversial biomarker for ICI treatment. Several single-arm, early phase clinical trials reported PD-L1 expression as a prognostic factor, but not in randomized trials. Several preliminary data in recent clinical trials suggest that patients with a high PD-L1 status have higher ORRs compared with those with a low PD-L1 status [52,53,54]. On the other hand, the IMvigor211 trial, which assessed the safety and efficacy of atezolizumab (anti-programmed-death-ligand 1 immune checkpoint inhibitor) versus chemotherapy for the treatment of locally advanced or metastatic UC after prior platinum-containing chemotherapy, showed that patients who received atezolizumab lived longer compared with patients who received chemotherapy, regardless of PD-L1 status [55]. In this study, however, PD-L1 immunohistochemistry appeared prognostic but not predictive. Also, the Keynote-045 trial showed that the PD-L1 combined score thresholds of 10% and 1% were not helpful as predictive biomarkers [4]. A systematic review, including 44 trials involving 6664 patients with solid tumors, showed a favorable predictive response of 2.26-fold higher in patients with PD-L1 expression compared with PD-L1-negative patients [56]. To understand the status and perspectives of the predictive response for ICI treatment in UC, three workshops were held from December 2018 to December 2019 [57]. The primary goal of these workshops was to develop recommendations for best approaches to PD-L1 testing in UC. One challenge with the use of PD-L1 immunohistochemistry is the different antibodies and scoring systems used for different agents. This makes the understanding of the role of PD-L1 more complicated, and it will be necessary to establish a uniform measurement system.

Recently, the TMB has been investigated as a promising biomarker to evaluate the response of ICI therapy. Somatic or germline mutations at the DNA level can lead to an increase in tumor-associated antigens, which results in high tumor immunogenicity. Although the relationship between the TMB and the response of ICI therapy has been reported in various types of malignancy, the data regarding UC are not fully elucidated [58,59,60]. Several studies have examined the impact of the TMB on the response to ICI therapy, but they were inconclusive, with some reporting a positive relationship and others a negative one [52,54]. As a representative study, the IMvigor211 trial showed that patients with a high TMB had longer overall survival in the atezolizumab cohort than the chemotherapy cohort [60]. Conversely, in a cohort of patients treated with atezolizumab in the IMvigor210 trial, there was no relationship between the TMB and clinical benefit [61]. Different methods (assays and cutoffs) may make data interpretation more difficult. Further validation and standardization are needed to elucidate the role of TMB for patients who receive ICI therapy.

Additionally, several studies have focused on the availability of circulating tumor DNA as a biomarker of multiple solid tumors. An analysis of circulating tumor DNA was performed in 29 patients treated with 6 weeks of durvalumab, and a significant reduction in circulating tumor DNA was observed in treatment responders but not in non-responders [7]. Similarly, Vandekerkhove et al. reported that a more aggressive form of disease in 104 patients with metastatic UC showed higher circulating tumor DNA levels [62]. Powles et al. evaluated outcomes in 581 UC patients who were evaluated for circulating tumor DNA from the IMvigor010 trial [63]. They showed that circulating tumor DNA testing at the start of therapy (cycle 1, day 1) identified 37% of the patients who were positive for circulating tumor DNA and who had a poor prognosis. Interestingly, the patients who were positive for circulating tumor DNA had improved disease-free survival and overall survival in the atezolizumab arm versus the observation arm, while no difference was observed in the disease-free survival and overall survival between the treatment arms for circulating tumor DNA negative patients. Further investigations on the role of circulating tumor DNA level as a predictive biomarker in UC patients are required.

The present study has several limitations, including its retrospective nature, the involvement of only two centers, and the small number of patients analyzed. In addition, a significant limitation of the analysis was not performing a shotgun metagenomic analysis of fecal samples to evaluate changes in the intestinal bacterial species and bacterial gene function profiles. Future studies of antibiotic classes, the duration of antibiotic exposure, and analysis of fecal samples are warranted to better understand the association between antibiotic exposure and ICI outcomes. Prospectively validated predictive biomarkers will provide valuable adjuncts to real-world clinical practice, but large trials with longer follow-up will be needed to clarify the many questions remaining.

## 5. Conclusions

In this retrospective study of 41 advanced UC patients receiving ICI treatment, poor ECOG-PS and high NLR were significantly associated with poor prognoses. Antibiotic exposure was not identified as a biomarker for ICI response. To clarify antibiotic exposure as a promising biomarker in real-world clinical practice, it will be necessary to determine whether the ICI response varies depending on the type of antibiotic and the duration of antibiotic exposure in future studies.

## Figures and Tables

**Table 1:** Characteristics of patients included in the analysis.

Variable	Patients (*n* = 41)
Age (years), median (range)	75 (58~81)
Observation period (months), median (range)	16.5 (1.0~47.8)
Male sex, *n* (%)	35 (85.4%)
Site of primary tumor, Upper urinary tract/Bladder, *n* (%)	16/25 (40%/60%)
Response criteria (RECIST), CR/PR/SD/PD, *n* (%)	7/5/2/29 (17%/12%/5%/70%)
ECOG-PS, 0/1/2, *n* (%)	27/10/4 (66%/24%/10%)
Number of prior regimens, 1/2/3/4, *n* (%)	20/14/6/1 (82%/8%/8%/2%)
Metastatic sites, liver/lung/bone/lymph node, *n* (%)	6/20/9/27 (15%/49%/22%/66%)
Number of metastatic organs, 1/2/3/4, *n* (%)	20/14/6/1 (48%/34%/15%/2%)
Hemoglobin, > 10 mg/dL/ < 10 ng/dL, *n* (%)	33/8 (80%/20%)
CRP baseline (mg/dL), median (range)	0.56 (0.03~21)
NLR baseline, median (range)	2.96 (1.27~28.4)
Antibiotic exposure, *n* (%)	16 (39%)
Duration of antibiotic exposure (days), median (range)	7 (7–30)
Antibiotic classes, Cephalosporin/fluoroquinolone/penicillin, *n* (%)	7/5/4 (44%/31%/25%)

**Table 2:** Overall response rate assessed with RECIST version 1.1.

Overall Response	*n* = 41 (%)
Complete response, *n* (%)	7 (17.1)
Partial response, *n* (%)	5 (12.2)
Stable disease, *n* (%)	2 (4.88)
Progressive disease, *n* (%)	27 (65.9)

**Table 3:** Adverse events in the treated population.

	Number of Patients (%)	
	Any Grade	Grade 3, 4, or 5
Any event	35 (85)	5 (12)
Event leading to treatment discontinuation	0	1 (2)
Event leading to death	0	1 (2)
Infusion reaction	2 (5)	2 (5)
Interstitial pneumonia	4(10)	1 (2)
Rash	9 (22)	
Liver dysfunction	2 (5)	1 (2)
Dysgeusia	1 (2)	
Fatigue	1 (2)	
Hypothyroidism	4 (10)	
Anorexia	2 (5)	
Leg edema	1 (2)	
Adrenal disorder	2 (5)	1 (2)
Isolated ACTH deficiency	2 (5)	
Parotiditis	1 (2)	
Constipation, diarrhea	2 (5)	
Melena	1 (2)	
Cutaneous sarcoidosis	1 (2)	

**Table 4:** Univariate and multivariate Cox’s regression analysis of PFS to clinicopathological features in platinum-resistant metastatic urothelial carcinoma patients treated with pembrolizumab.

		Univariate Analysis		Multivariate Analysis	
Factor	Category	HR (95%CI)	*p*-Value	HR (95%CI)	*p*-Value
Age (years)	<72 vs. ≥72	1.51 (0.72–3.21)	0.28		
Gender	Female vs. Male	1.07 (0.37–3.09)	0.90		
ECOG-PS	0.1 vs. ≥2	5.17 (1.62–16.5)	0.01	2.63 (0.80–8.73)	0.11
Surgical resection	No vs. Yes	0.58 (0.28–1.23)	0.16		
Any irAEs	Negative vs. Positive	0.72 (0.35–1.49)	0.38		
Neutrophil-to-lymphocyte ratio (NLR)	≤3.0 vs. >3.0	3.01 (1.41–6.42)	0.01	1.97 (0.85–4.57)	0.12
Hb (g/dL)	≤11 vs. >11	2.70 (1.26–5.75)	0.01	1.89 (0.84–4.57)	0.13
CRP (mg/dL)	≤1.0 vs. >1.0	1.20 (0.58–2.47)	0.63		
Tumor site	Lower vs. Upper	0.43 (0.20–0.94)	0.03	0.57 (0.25–1.26)	0.16
Site of metastasis	Bone	1.14 (0.49–2.66)	0.76		
	Lymph node	1.07 (0.50–2.29)	0.87		
	Lung	1.07 (0.52–2.18)	0.86		
	Liver	0.89 (0.31–2.57)	0.83		
Number of metastases	<1 vs. ≥2	1.26 (0.61–2.59)	0.53		
Antibiotics prior to pembrolizumab administration	No vs. Yes	1.16 (0.53–2.54)	0.71		

**Table 5:** Univariate and multivariate Cox’s regression analysis of OS to clinicopathological features in platinum-resistant metastatic urothelial carcinoma patients treated with pembrolizumab.

		Univariate Analysis		Multivariate Analysis	
Factor	Category	HR (95%CI)	*p*-Value	HR (95%CI)	*p*-Value
Age (years)	<72 vs. ≥72	2.25 (0.93–5.40)	0.07		
Gender	Female vs. Male	1.19 (0.36–3.98)	0.78		
ECOG-PS	0.1 vs. ≥2	20.4 (4.30–96.9)	0.01	6.33 (1.24–32.3)	0.03
Surgical resection	No vs. Yes	0.49 (0.22–1.08)	0.08		
Any irAEs	Negative vs. Positive	0.81 (0.37–1.79)	0.60		
Neutrophil-to-lymphocyte ratio (NLR)	≤3.0 vs. >3.0	3.53 (1.49–8.36)	0.01	2.79 (1.07–7.23)	0.04
Hb (g/dL)	≤11 vs. >11	3.38 (1.47–7.95)	0.01	2.35 (0.94–5.90)	0.07
CRP (mg/dL)	≤1.0 vs. >1.0	2.34 (1.06–5.17)	0.04	2.24 (0.92–5.46)	0.07
Tumor site	Lower vs. Upper	0.45 (0.19–1.08)	0.07		
Site of metastasis	Bone	2.37 (0.95–5.76)	0.06		
	Lymph node	1.69 (0.70–4.05)	0.24		
	Lung	1.04 (0.47–2.28)	0.92		
	Liver	1.25 (0.43–3.64)	0.69		
Number of metastases	<1 vs. ≥2	2.27 (1.01–5.07)	0.06		
Antibiotics prior to pembrolizumab administration	No vs. Yes	1.68 (0.74–3.80)	0.21		

## Data Availability

Data available on request due to privacy or ethical restrictions. The data presented in this study are available on request from the corresponding author. The data are not publicly available due to institutional policy.

## Abbreviations

AEs	Adverse events
BSC	best supportive care
CD4	cluster of differentiation 4
CD8	cluster of differentiation 8
CR	complete response
CRP	C-reactive protein
CTCAE	common terminology criteria for adverse events
DNA	deoxyribonucleic acid
ECOG-PS	Eastern Cooperative Oncology Group performance status
FN	febrile neutropenia
GC	gemcitabine, cisplatin
ICI	immune checkpoint inhibitor
IgG4κ	immunoglobulin G4κ
MVAC	methotrexate, vinblastine, doxorubicin, and cisplatin
NLR	neutrophil-to-lymphocyte ratio
ORR	objective response rate
OS	overall survival
PD	progressive disease
PD-L1	programmed death ligand-1
PFS	progression-free survival
PR	partial response
PS	performance status
RECIST	response evaluation criteria in solid tumors
SD	stable disease
SPSSs	Statistical Package for the Social Sciences
TMB	tumor mutational burden
UC	urothelial carcinoma
